# Applications of the pipeline environment for visual informatics and genomics computations

**DOI:** 10.1186/1471-2105-12-304

**Published:** 2011-07-26

**Authors:** Ivo D Dinov, Federica Torri, Fabio Macciardi, Petros Petrosyan, Zhizhong Liu, Alen Zamanyan, Paul Eggert, Jonathan Pierce, Alex Genco, James A Knowles, Andrew P Clark, John D Van Horn, Joseph Ames, Carl Kesselman, Arthur W Toga

**Affiliations:** 1 Laboratory of Neuro Imaging (LONI), University of California, Los Angeles, Los Angeles, 90095, USA; 2 Biomedical Informatics Research Network (BIRN), Information Sciences Institute, University of Southern California, Los Angeles, 90292, USA; 3 Departments of Psychiatry and Human Behavior, University of California, Irvine, Irvine, 92617, USA; 4 Department of Computer Science, University of California, Los Angeles, Los Angeles, 90095, USA; 5 Zilkha Neurogenetic Institute, USC Keck School of Medicine, Los Angeles, 90033, USA

## Abstract

**Background:**

Contemporary informatics and genomics research require efficient, flexible and robust management of large heterogeneous data, advanced computational tools, powerful visualization, reliable hardware infrastructure, interoperability of computational resources, and detailed data and analysis-protocol provenance. The Pipeline is a client-server distributed computational environment that facilitates the visual graphical construction, execution, monitoring, validation and dissemination of advanced data analysis protocols.

**Results:**

This paper reports on the applications of the LONI Pipeline environment to address two informatics challenges - graphical management of diverse genomics tools, and the interoperability of informatics software. Specifically, this manuscript presents the concrete details of deploying general informatics suites and individual software tools to new hardware infrastructures, the design, validation and execution of new visual analysis protocols via the Pipeline graphical interface, and integration of diverse informatics tools via the Pipeline eXtensible Markup Language syntax. We demonstrate each of these processes using several established informatics packages (e.g., miBLAST, EMBOSS, mrFAST, GWASS, MAQ, SAMtools, Bowtie) for basic local sequence alignment and search, molecular biology data analysis, and genome-wide association studies. These examples demonstrate the power of the Pipeline graphical workflow environment to enable integration of bioinformatics resources which provide a well-defined syntax for dynamic specification of the input/output parameters and the run-time execution controls.

**Conclusions:**

The LONI Pipeline environment http://pipeline.loni.ucla.edu provides a flexible graphical infrastructure for efficient biomedical computing and distributed informatics research. The interactive Pipeline resource manager enables the utilization and interoperability of diverse types of informatics resources. The Pipeline client-server model provides computational power to a broad spectrum of informatics investigators - experienced developers and novice users, user with or without access to advanced computational-resources (e.g., Grid, data), as well as basic and translational scientists. The open development, validation and dissemination of computational networks (pipeline workflows) facilitates the sharing of knowledge, tools, protocols and best practices, and enables the unbiased validation and replication of scientific findings by the entire community.

## I. Background

Contemporary informatics and genomic research require efficient, flexible and robust management of large heterogeneous data [1,2], advanced computational tools [3], powerful visualization [4], reliable hardware infrastructure [5], interoperability of computational resources [6,7], and provenance of data and protocols [8-10]. There are several alternative approaches for high-throughput analysis of large amounts of data such as using various types of shell-scripts [11,12] and employing tool-specific graphical interfaces [13-15]. The large-scale parallelization, increased network bandwidth, need for reproducibility, and wide proliferation of efficient and robust computational and communication resources are the driving forces behind this need for automation and high-throughput analysis. There is a significant push to increase and improve resource development, enable the expansion of integrated databases and vibrant human/machine communications, and increase the distributed grid and network computing bandwidth [16,17]. To meet these needs we present a new visual language programming framework for genomics and bioinformatics research based on heterogeneous graphical workflows. We demonstrate the construction, validation and dissemination of several analysis protocols using a number of independent informatics and genomics software suites - miBLAST [18], EMBOSS [19], mrFAST [20], GWASS [21], MAQ [22], SAMtools [23], Bowtie [24,25], etc. These types of genomics and informatics tools were chosen as they span a significant component of the informatics research and, at the same time, they have symbiotic relations which enable their interoperability and integration.

The continual evolution and considerable variability of informatics and genomics data, software tools and web-service present challenges in the design, management, validation and reproducibility of advanced biomedical computing protocols. There are a number of graphical environments for visual design of computational protocols (pipelines), tool integration, interoperability and meta-analysis [15,26]. Most of them aim to enable new types of analyses, facilitate new applications, promote interdisciplinary collaborations, and simplify tool interoperability [27]. Compared to other environments, the Pipeline offers several advantages, including a distributed grid-enabled, client-server, and fail-over-safe infrastructure, quick embedding of new tools within the Pipeline computational library, and efficient dissemination of new protocols to the community. Table 1 provides a summary of the synergies and differences between the LONI Pipeline and several alternative graphical workflow environments. Additional comparisons between the LONI Pipeline and various alternative environments for software tool integration and interoperability are presented here [15].

**Table 1:** Comparison of common graphical workflow environments

Workflow Environment	Requires Tool Recompiling	Data Storage	Platform Independent	Client-Server Model	Grid Enabled	Application Area	URL
LONI Pipeline [15]	N	External	Y	Y	Y (DRMAA)	Area agnostic	http://Pipeline.loni.ucla.edu

Taverna [35]	Y (via API)	Internal (MIR)	Y	N	Y (myGRID)	Bioinformatics	http://www.taverna.org.uk

Kepler [14]	Y (via API)	Internal (actors)	Y	N	Y (Ecogrid)	Area agnostic	http://kepler-project.org

Triana [36]	Y	Internal data structure	Y	N	Y (gridLab)	Hetero-geneous Apps	http://www.trianacode.org

Galaxy [37]	N	External	N (Linux, Mac)	Y	N (Cloud EC2)	Bioinformatics	http://usegalaxy.org

Pipeline Pilot	Y	Internal	Y	N	N	Biochemistry	http://accelrys.com/products/pipeline-pilot/

AVS [38]	Y	Internal	Y (platform build)	N	N	Advanced Visualization	http://www.avs.com

VisTrails [39]	Y	Internal	N	N	N	Scientific Visualization	http://www.vistrails.org

Bioclipse [40]	N (plug-ins)	Internal	Y	N	N	Biochemistry Bioinformatics	http://www.bioclipse.net

Y = yes, N = no; Taverna MIR plug-in, MIR = myGrid Information Repository; DRMAA = Distributed Resource Management Application API.

**Figure 1:**
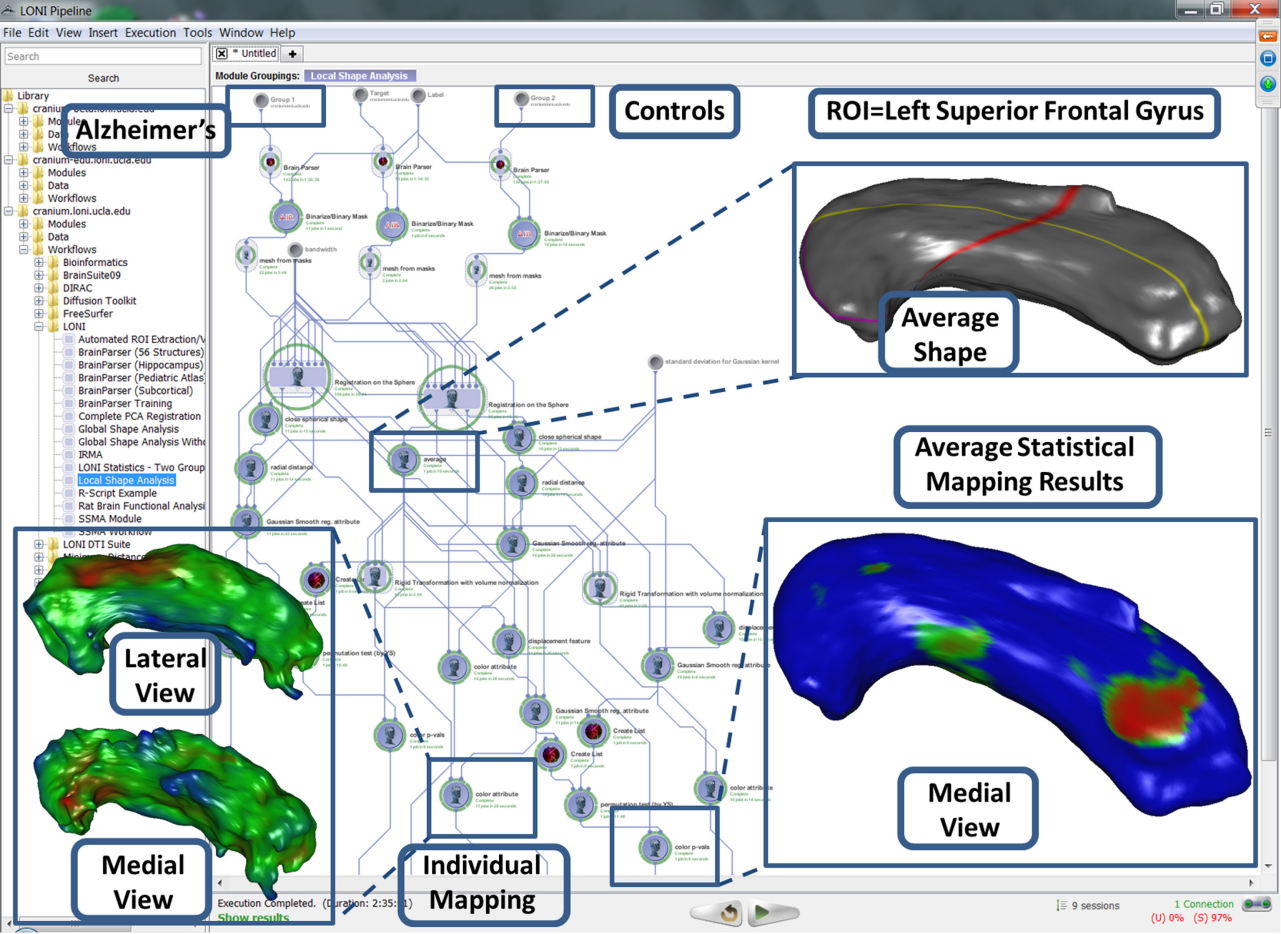
**An example of a completed Pipeline workflow (Local Shape Analysis) representing an end-to-end computational solution to a specific brain mapping problem**. This pipeline protocol starts with the raw magnetic resonance imaging data for 2 cohorts (11 Alzheimer's disease patients and 10 age-matched normal controls). For each subject, the workflow automatically extracts a region of interest (left superior frontal gyrus, LSFG. using BrainParser [1]) and generates a 2D shape manifold model of the regional boundary [2,3]. Then the pipeline computes a mean LSFG shape using the normal subjects LSFG shapes, coregisters the LSFG shapes of all subjects to the mean (atlas) LSFG shape, and maps the locations of the statistically significant differences of the 3D displacement vector fields between the 2 cohorts. The insert images illustrate the mean LSFG shape (top-right), the LSFG for one subject (bottom-left), and the between-group statistical mapping results overlaid on the mean LSFG shape (bottom-right), red color indicates p-value < 0.01.

Previously, we have demonstrated a number of imaging [15,28], brain mapping [29,30] and neuroscientific [31,32] applications using the Pipeline environment. One shape morphometry analysis protocol, using BrainParser [33] several shape manifold models of regional boundary [2,3] implemented via the Pipeline environment is illustrated on Figure 1. This is a complete neuroimaging solution (available within the Pipeline core computational library) which automatically extracts, models and statistically analyzes local and regional shape differences between several cohorts based on raw magnetic resonance imaging data. The **Results **section below includes hands-on examples demonstrating specific informatics and genomics computing protocols for sequence data analysis and interoperability of heterogeneous bioinformatics resources.

## II. Implementation

As an external inter-resource mediating layer, the Pipeline environment [15] utilizes a distributed infrastructure model for mediating disparate data resources, software tools and web-services. No software redesign or rebuilding modifications of the existing resources are necessary for their integration with other computational components. The Pipeline eXtensible Markup Language (XML) schema enables the inter-resource communication and mediation layer. Each XML resource description contains important information about the tools location, the proper invocation protocol (i.e., input/output types, parameter specifications, etc.), run-time controls and data-types. The Pipeline XML schema http://pipeline.loni.ucla.edu/support/xml-overview/ also includes auxiliary metadata about the resource state, specifications, history, authorship, licensing, and bibliography. Using this resource metadata, the Pipeline infrastructure facilitates the integration of disparate resources and provides a complete and comprehensive protocol provenance [15] for the data, tools, hardware and results. Individual module descriptions and entire protocol XML objects are managed as .PIPE files, facilitate the broad dissemination of resource metadata descriptions via web services, and promote constructive utilization of multidisciplinary tools and expertise by professionals, novice users and trainees.

In this paper we demonstrate the concrete details for utilizing several informatics and genomics suites of tools and show how such disparate resources may be integrated within the Pipeline environment. Many additional resources (data, tools and protocols) are also available within the core Pipeline computational library (e.g., R statistical computing, image analysis, and statistical inference) and more can easily be added to (local or remote) Pipeline server libraries following the protocols described below. In addition to presenting a number of bioinformatics and genomics applications using the Pipeline environment, this manuscript described some improvements of Pipeline version 5.2 over previous versions [15], e.g., enhanced user-management, client-server pipeline web-start (PWS) interface, directory access control, and improved Java authentication and authorization service interface.

### Pipeline Architecture

**Figure 2:**
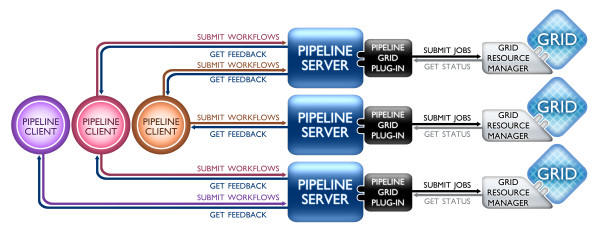
**High-level schematic representation of the communication between multiple local Pipeline clients connected to multiple remote Pipeline servers**.

The Pipeline software architecture design is domain and hardware independent which makes the environment useful in different computational disciplines and on diverse hardware infrastructures. The Pipeline environment may be utilized in three synergistic mechanisms [15]. The first one http://pipeline.loni.ucla.edu/downloads involves the local use of the Pipeline client via connection to a remote server running natively on a hardware system which includes all appropriate plug-ins for system-specific grid managers, file systems, network, and communication protocols. The second type of Pipeline server distribution relies on virtualization technology. The virtualized Pipeline infrastructure provides end-users with the latest stable pre-compiled environment including all pre-installed open-source informatics tools. The resulting Pipeline Virtual Environment (PNVE, http://pipeline.loni.ucla.edu/PNVE), contains the complete self-contained execution environment that can be run locally or on remote grid computing environment. Because the Pipeline virtualization environment tightly mirrors that of the LONI grid http://www.loni.ucla.edu/twiki/bin/view/Infrastructure/GridComputing, users gain access to all LONI image processing, brain mapping and informatics tools and services. Version 1.0 of the virtual Pipeline environment is based on Ubuntu http://www.ubuntu.com and VMware http://www.vmware.com technologies. The third Pipeline distribution mechanism is called Distributed Pipeline Server (DPS, http://pipeline.loni.ucla.edu/DPS). This distribution includes a user-friendly graphical user interface (GUI) for automated native system configuration, installation and deployment of the Pipeline server, the available XML computational library, and back-end software tools. The Pipeline environment uses a client-server architecture, but each Pipeline client may also act as a local server and manage job submission and execution. Following proper authentication, the process of a client submitting a workflow for execution to a specified server prompts the server to translate (break) the workflow into parallel jobs and send them to the grid resource manager which in turn farms these to the back-end grid (or multiple cores). When a job is complete, the server retrieves the results from the grid resource manager and sends out subsequent jobs from the active workflow. The client receives status updates from the server at regular intervals, Figure 2. Currently, the Pipeline server supports Distributed Resource Management Application API (DRMAA, http://www.DRMAA.org) interface and Java Gridengine Database Interface (JGDI) to communicate to the grid resource manager. These include many of the popular grid resource managers, including Sun/Oracle Grid Engine http://en.wikipedia.org/wiki/Oracle_Grid_Engine, GridWay http://www.gridway.org, PBS/Torque http://www.ClusterResources.com.

### Software Implementation

The entire Pipeline source code is in Java, including the client, server, network, execution and database components. The Pipeline server has a grid plug-in component, which provides communication between the Pipeline server and grid resource manager. In addition to JGDI and DRMAA support, it supports custom plug-in of grid resource managers. The API for grid plug-in is available on the Pipeline website http://pipeline.loni.ucla.edu/support/server-guide/pipeline-grid-plugin-api-developers-guide/.

## II.1 Software Development

### User-management

As the hardware resources (e.g., storage, CPU, memory) are generally limited and the available Pipeline servers are finite, a handful of heavy users may disproportionately monopolize the Pipeline services and the underlying hardware infrastructure. To prevent this from occurring, a fair-usage policy is implemented to manage the sharing of these limited resources. This policy, referred to as "User Management," ensures that no users may utilize more than a predefined percent (which is specified as a preference by the server administrator) of the available resources at a fixed time. Different pipeline servers may have different usage percentage values. The number submissions allowed is dynamic and changes after each new job is submitted, as it depends on the number of available nodes/slots and the number of user-specific jobs already scheduled. For instance, a running Pipeline server which has only 100 slots available where the limit percent value is set to 50 would allow the first user to utilize no more than 50 slots. The Pipeline user-manager calculates the number of slots the user can use with the following formula:

where: **T **is the total number of available slots, **U **is the number of currently used slots by all the users, **UC **is the number of used slots by current user only, and **P **is the limit percent value specified by the Pipeline server administrator. This user management protocol significantly improves the server usability and allows each user to submit at least part of their jobs in real time, although it slightly reduces the optimal usage of the server.

### Directory Access Control

The Pipeline server allows administrator control over the user access to binary executables, workflows/modules and server files (browseable via the Pipeline Remote File Browser GUI). Server administrators may also specify a list of users who can, or cannot, access a list of directories. This is a convenient feature when a Pipeline server supports several different categories of groups of users and some server files should not be visible or executable by various users or groups.

### User Authentication using JAAS

The Pipeline authenticates users using the Java Authentication and Authorization Service http://java.sun.com/javaee/security/, which allows the server operator to authenticate usernames and passwords against any type of system. When a user connects to a Pipeline server, the Pipeline tries to create a new JAAS Object called LoginContext and if the creation is successful, attempts to call the object's login method. If the method returns "true", then the Pipeline allows the user to continue. Otherwise the user is disconnected from the server with an "Authentication rejected" message.

### File Permission on Shared Temporary Directories

Each Pipeline server has one directory where all the temporary files are stored. Files located in this directory need to be accessible only by the Pipeline server administrator and by the user who started the workflow. Pipeline server has a special flag in its preferences file which enables this feature and creates special permissions for each file in the temporary directory, which enables safe and secure management of files in the temporary directory.

### Stability

The performance of the Pipeline server (V.5.0+) has significantly improved over V.4 [34] in terms of client-server communication, server reliability and stability. This was accomplished by introducing architectural changes, bug-fixes (e.g., resolving memory leaks), and additional new features like failover, Grid plug-ins and array job-submission. **Failover**: The server failover feature improves robustness and minimizes service disruptions in the case of a single Pipeline server failure. It is available on servers running Linux or other UNIX operating systems. The core of failover is running two actual Pipeline servers in parallel, a primary and a secondary, a virtual Pipeline Server name, and de-coupling and running the persistence database on a separate system. Each of the two servers monitors the state of its counterpart. In the event that the primary server with the virtual Pipeline server name has a catastrophic failure, the secondary server will assume the virtual name, establish a connection to the persistence database, take ownership of all current Pipeline jobs dynamically, and restart the formerly primary server as secondary. **Grid plug-ins**: This feature allows Pipeline to run a new Grid plug-in process instead of attaching the plug-in to the actual Pipeline process. This makes Grid plug-ins and resource management libraries isolated from the Pipeline server and prevents server crashes if any of the lower level libraries crash. After having this feature, the server up time has been dramatically increased. **Array Jobs**: The Pipeline server now supports array job submission which improves the total processing time of a workflow by combining repeated jobs into one array job. Each job in the array has its own output stream and error stream. This feature increases the speed of job submission especially for modules with multiple instances (e.g., 100-10,000 subjects). Depending of the module's number of instances, there is 10-65% speed improvement when using array jobs versus sequential individual job submissions. This feature is configurable and server administrator may set preferences about how array jobs will be submitted on each Pipeline server.

## II.2 Implementation of Genomics and Informatics Pipeline Protocols

The following steps are necessary to develop a complete Pipeline biomedical solution to a well-defined computational challenge - *protocol design, tool installation, module definition, workflow implementation, workflow validation*, and *dissemination *of the resulting workflow for broader community-based testing and utilization. Each of these steps is described in detail below and screencasts and videos demonstrating these steps are available online http://pipeline.loni.ucla.edu/support/.

### Protocol design

**Figure 3:**
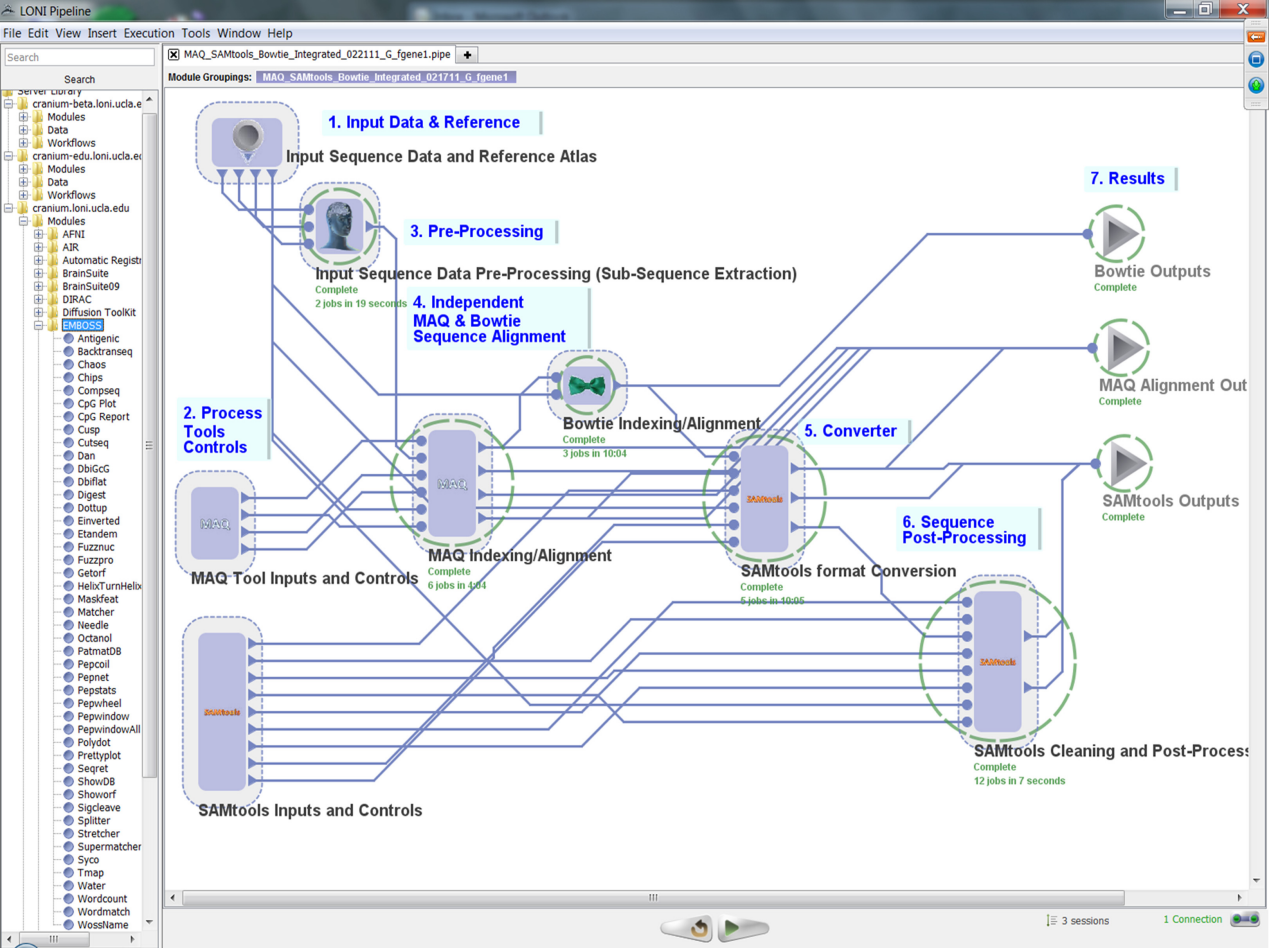
**A high-level group-folded representation of the alignment and assembly protocol, Table 2, as a Pipeline graphical workflow**.

This is the most important step in the design of a new Pipeline graphical workflow to solve a specific informatics or genetics problem and typically involves multidisciplinary expert users with sufficient scientific and computational expertise. In practice, most genomics challenges may be approached in several different ways and the final Pipeline XML workflow will greatly depend on this initial step. In this step, it may be most appropriate to utilize a top-down approach for outlining the general classes of sequence data analysis, then the appropriate sub-classes of analyses, specific tools, test-data, invocation of concrete tools, and a detailed example of executable syntax for each step in the protocol. Below is a hierarchical example of a discriminative design for a sequence alignment and assembly protocol demonstrated in the **Results **section. These steps are not different from other approaches for developing and validating informatics and genomics protocols, however the explicit hierarchical formulation of these steps is only done once by the expert user(s). All subsequent protocol redesigns, modifications and extensions may be accomplished (by all types of users) directly on the graphical representation of the protocol with the Pipeline graphical user interface (GUI). Table 2 contains an example of the specification of an alignment and assembly protocol, which is also demonstrated as a complete Pipeline genomics solution on Figure 3.

**Table 2:** An example of a hierarchical alignment and assembly protocol specification

***Alignment and Assembly***
*A preprocessing step*: Extracting a sub-sequence of the genomic sequence. This step is not required, but may be useful for some preliminary tests and protocol validation. It restricts the size of the sequences and expedites the computation
*Input*: reads files output of Illumina sequencing pipeline (sequence.txt files)
*Tool*: LONI Sub-Sequence extractor
*Server Location*:/projects1/idinov/projects/scripts/extract_lines_from_Textfile.sh
*Output*: Shorter sequence.fastq file
*Data conversion*: File conversion of solexa fastq in sanger fastq format
*Input*: reads files output of Illumina sequencing pipeline (sequence.txt files)
*Tool*: MAQ (sol2sanger option): Mapping and Assembly with Quality
*Server Location*:/applications/maq
*Output*: sequence.fastq file
*Binary conversion*: Conversion of fastq in a binary fastq file (bfq)
*Input*: sequence.fastq file
*Tool*: MAQ (fastq2bfq option)
*Server Location*:/applications/maq
*Output*: sequence.bfq file
*Reference conversion*: Conversion of the reference genome (fasta format) in binary fasta
*Input*: reference.fasta file (to perform the alignment)
*Tool*: MAQ (fasta2bfa option)
*Server Location*:/applications/maq
*Output*: reference.bfa file
*Sequence alignment*: Alignment of data sequence to the reference genome
*Using MAQ:*
*Input*: sequence.bfq, reference.bfa
*Tool*: MAQ (map option)
*Server Location*:/applications/maq
*Output*: alignment.map file
*Using Bowtie:*
*Input*: reference.fai, sequence.bfq,
*Tool*: Bowtie (map option)
*Server Location*:/applications/bowtie
*Output*: alignment.sam file
*Indexing*: Indexing the reference genome
*Input*: reference.fa
*Tool*: samtools (faidx option)
*Server Location*:/applications/samtools-0.1.7_x86_64-linux
*Output*: reference.fai
*Mapping conversion:*
*MAQ2SAM*:
*Input*: alignment.map file
*Tool*: samtools (maq2sam-long option)
*Server Location*:/applications/samtools-0.1.7_x86_64-linux
*Output*: alignment.sam file
*SAM to full BAM*:
*Input*: alignment.sam, reference.fai file
*Tool*: samtools (view -bt option)
*Server Location*:/applications/samtools-0.1.7_x86_64-linux
*Output*: alignment.bam file
*Removal of duplicated reads*:
Input: alignment.bam file
Tool: samtools (rmdup)
Server Location:/applications/samtools-0.1.7_x86_64-linux
Output: alignment.rmdup.bam file
*Sorting*:
*Input*: alignment. rmdup.bam file
*Tool*: samtools (sort option)
*Server Location*:/applications/samtools-0.1.7_x86_64-linux
*Output*: alignment. rmdup.sorted.bam file
*MD tagging*:
*Input*: alignment. rmdup.sorted.bam file and reference REF.fasta file
*Tool*: samtools (calmd option)
*Server Location*:/applications/samtools-0.1.7_x86_64-linux
*Output*: alignment. rmdup.sorted.calmd.bam file
*Indexing*:
*Input*: alignment.rmdup.sorted.calmd.bam file
*Tool*: samtools (index option)
*Server Location*:/applications/samtools-0.1.7_x86_64-linux
*Output*: alignment. rmdup.sorted.calmd.bam.bai file

This protocol is implemented as a Pipeline graphical workflow and demonstrated in the Results section. Figure 3 shows the corresponding Pipeline graphical workflow implementing this genomics analysis protocol.

### Tool installation

Once the protocol is finalized, the workflow designer and the administrator of the Pipeline server need to ensure that all tools (which are not already deployed with the Pipeline server installation) are available at the specified server locations. Note that tools are installed in specific locations which may be varying for different hardware platforms and sites. The Pipeline Library manager http://pipeline.loni.ucla.edu/support/server-guide/configuration/ facilitates the portability of the XML-based Pipeline graphical workflows by defining a hash-map between different software suites, computational tools, versions and executable locations. Thus a well-defined Pipeline graphical workflow only references the software suite, its version and the specific tool necessary for the specific computational task. The Pipeline server then interprets, maps and constructs the specific executable commands which are relayed to the grid manager as concrete jobs and scheduled for execution.

### Module definition

The module definition is accomplished via the Pipeline GUI. Each of the executable processes needs to be described individually and independently as a node (or a module) in the workflow graph http://pipeline.loni.ucla.edu/support/user-guide/creating-modules/. This step also includes the independent testing and validation of the execution of each of the individual nodes (modules) using appropriate data. The result of this module definition step is an XML file (*.pipe) representing the tool invocation syntax, which can be broadly used, shared, modified, extended and integrated with other module descriptions to form complex graphical workflow protocols.

### Workflow implementation

This protocol skeletonization process is important as it lays out the flow of the data and indicates the complete data analysis provenance http://pipeline.loni.ucla.edu/support/user-guide/building-a-workflow/. After all necessary modules are independently defined and validated, we need to integrate them into a coherent and scientifically-valid pipeline workflow. Frequently the developer(s) makes use of module groupings to abstract computational complexity, conditional and looping modules to direct the processing flow, and specify data sources (inputs) and data sinks (results). In addition, workflow-wide and module-specific meta-data documentation is also provided in this step. These include appropriate workflow preferences, variables, references/citations (using PMCIDs), URLs, licenses, required vs. optional parameters, inputs, outputs and run-time controls, etc.

### Workflow validation

The workflow validation step involves the testing and fine-tuning of the implemented workflow to ensure the results of each intermediate step as well as the final results are reasonable, appropriately captured and saved, and tested against alternative protocols (e.g., outputs of analogous scripts). This automated workflow validation step ensures that input datasets and the output results are well-defined, verifies the provenance information about the pipeline workflow, as well as provides user documentation, limitations, assumptions, potential problems and solutions, usability and support annotation.

### Dissemination

Validated pipeline workflows may be disseminated as XML documents via email, web-sites and pipeline server libraries, as well as Biositemaps objects [27] in XML or RDF format http://www.Biositemaps.org. Any valid pipeline workflow may be loaded by any remote Pipeline client. However, execution of a particular workflow may require access to a Pipeline server where all tools referenced in the workflow are available for execution, the user has the appropriate credentials to access the remote Pipeline servers, data, software tools and services. In addition, some minor workflow modifications may be necessary prior to the execution of the pipeline workflow (e.g., server changes, data input and result output specifications, re-referencing to executable tools, variable specifications, review of protocol documentation, etc.) Although, each Pipeline client is itself a server, typical users would not run workflows on the same (client) machine but rather remotely login to a Pipeline server to outsource the computing-intensive tasks. Pipeline clients can disconnect and reconnect frequently to multiple Pipeline servers to submit workflows and monitor the status of running workflows in real time.

## III. Results and Discussion

We now demonstrate the complete process of installing, XML-wrapping (metadata describing), employing and integrating tools from several informatics software suites - **miBLAST **(Basic Local Alignment Search Tool for nucleotide sequence queries) [18], **EMBOSS **(European Molecular Biology Open Software Suite) [19], **mrFAST **(micro-read Fast Alignment Search Tool) [20], **GWASS **(Genome-Wide Association Study Software) [21], **MAQ **(Mapping and Assembly with Qualities) [22], **SAMtools **(Sequence Alignment and Mapping Tools) [23], and **Bowtie **[24,25]. Each of these packages includes a large number of tools and significant capabilities. The Pipeline XML module definitions for some of the tools within these packages may not yet be implemented, or may be incompletely defined within the Pipeline library. However, following this step-by-step guideline, the entire community may extend and improve the existing, as well as describe and distribute additional, Pipeline XML module wrappers for other tools within these suites. Additional genomics and informatics suites and resources may also be similarly described in the Pipeline XML syntax and made available to the community, as needed.

## III.1 miBLAST

◦ **URL**: http://www.eecs.umich.edu/~jignesh/miblast/

◦ **Description**: miBLAST is a tool for efficiently BLASTing a batch of nucleotide sequence queries. Such batch workloads contain a large number of query sequences (for example, consider BLASTing a library of oligonucleotide probe set against an EST database, http://www.ncbi.nlm.nih.gov/nucest). These batch workloads can be evaluated by BLASTing each individual query one at time, but this method is very slow for large batch sizes.

◦ **Installation**: The downloading, installation and configuration of the miBLAST suite on Linux OS kernel takes only 10 minutes following these instructions: http://www.eecs.umich.edu/~jignesh/miblast/installation.html.

◦ **Pipeline Workflow**

▪ *XML Metadata description*: The module descriptions for each of the nodes in the Pipeline workflow took about 30 minutes each. The design of the complete workflow took 8 hours because this suite generates many implicit outputs and is intended to be run each time from the core build directory. This presents a challenge for multiple users using the same routines and generating the same implicit filenames (results). To circumvent this problem, we added several auxiliary modules in the beginning (to copy the entire distribution to a tem space) and at the end (to clean up the intermediate results). Notice that wrapper shell-scripts had to also be developed to address the problems with implicit output filenames. Finally, flow-of-control connections, in addition to standard data-passing connections, were utilized to direct the execution of the entire protocol.

▪ *Name*: miBLAST_Workflow.pipe

▪ *URL*: http://www.loni.ucla.edu/twiki/bin/view/CCB/PipelineWorkflows_BioinfoBLAST

**Figure 4:**
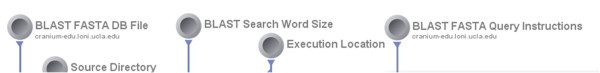
**A snapshot of the input parameters (data-sinks) for the miBLAST Pipeline workflow**.

**Figure 5:**
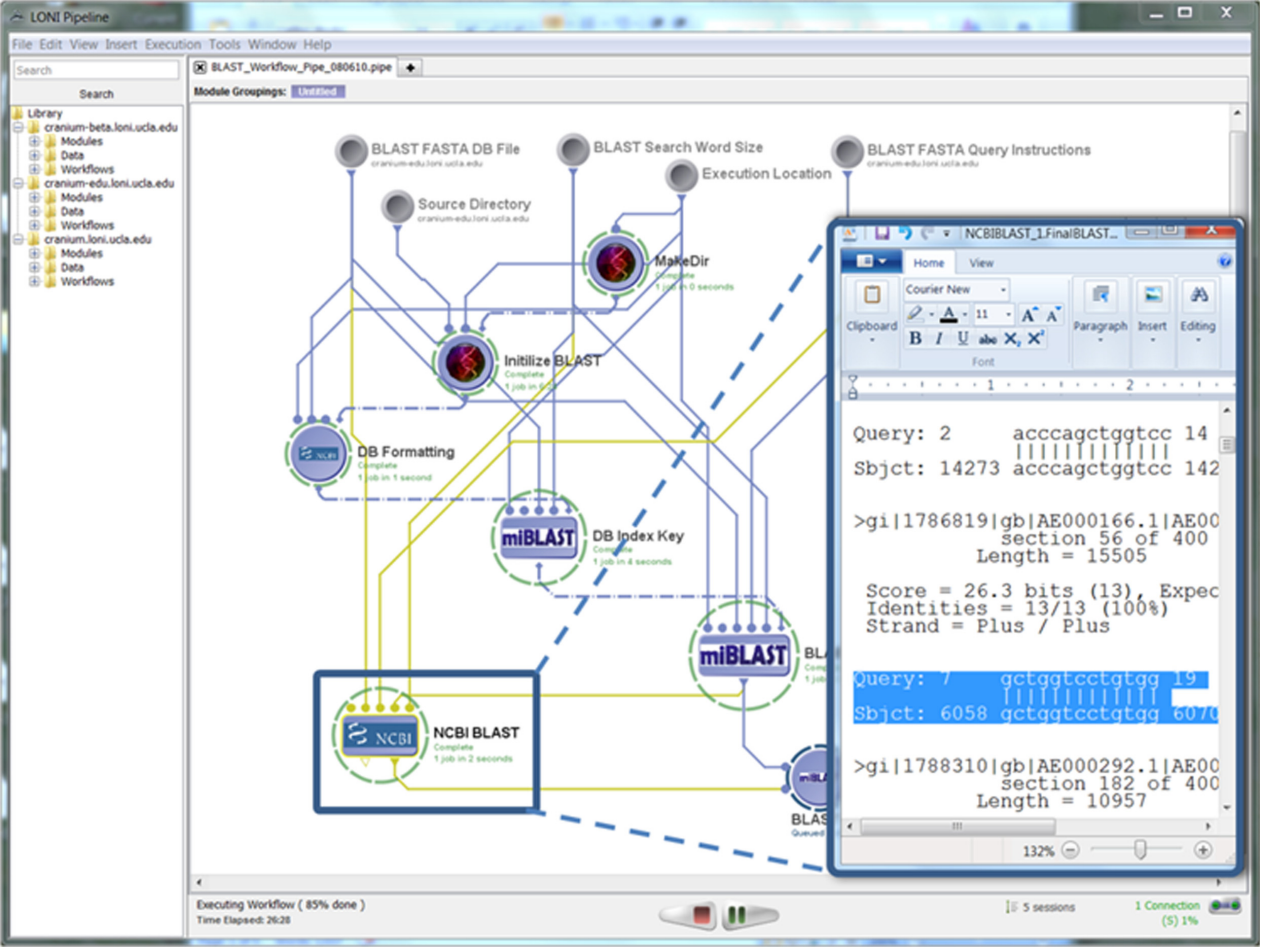
**A snapshot of the completed miBLAST Pipeline workflow**. The insert image illustrates the final output result, see Table 3.

▪ *Screenshots*:

• Input: Figure 4 shows a snapshot of the input parameters (data-sources) for the corresponding Pipeline workflow.

• Pipeline Execution: Figure 5 shows the completed miBLAST pipeline workflow and a fragment of the output alignment result.

• Output: Table 3 contains the beginning of the output result from the miBLAST workflow.

**Table 3:** A fragment of the output result from the miBLAST pipeline workflow, see Figure 5

BLASTN 2.2.8 [Jan-05-2004]		
		
Reference: Altschul, Stephen F., Thomas L. Madden, Alejandro A. Schaffer, Jinghui Zhang, Zheng Zhang, Webb Miller, and David J. Lipman (1997), "Gapped BLAST and PSI-BLAST: a new generation of protein database search programs", Nucleic Acids Res. 25:3389-3402.		
		
Query=probe:HG-U133A:1007_s_at:467:181; Interrogation_Position=3330; Antisense;		
(25 letters)		
		
Database:/ifs/ccb/CCB_SW_Tools/others/Bioinformatics/Blast/miBLAST/miblast/src/example/ecoli.nt		
400 sequences; 4,662,239 total letters		
		
Searching.done		
		
Sequences producing significant alignments:	Score (bits)	E Value
		
gi|1790777|gb|AE000503.1|AE000503 Escherichia coli K-12 MG1655 s...	26	0.69
gi|2367246|gb|AE000436.1|AE000436 Escherichia coli K-12 MG1655 s...	26	0.69
gi|1788338|gb|AE000294.1|AE000294 Escherichia coli K-12 MG1655 s...	26	0.69
		
gi|1786819|gb|AE000166.1|AE000166 Escherichia coli K-12 MG1655 s...	26	0.69
gi|1788310|gb|AE000292.1|AE000292 Escherichia coli K-12 MG1655 s...	24	2.7

▪ *Approximate time to complete*: 20-30 minutes.

## III.2 EMBOSS

◦ **URL**: http://EMBOSS.sourceforge.net/

◦ **Tool**: EMBOSS *Matcher*

◦ **Description**: Finds the best local alignments between two sequences. It can be used to compare two sequences looking for local sequence similarities using a rigorous algorithm.

◦ **Installation**: The downloading, installation and configuration of the entire EMBOSS suite on Linux OS kernel takes 15 minutes following these instructions: http://emboss.sourceforge.net/docs/faq.html.

◦ **Pipeline Workflow**

▪ *XML Metadata description*: The Pipeline XML for a number of EMBOSS tools are available. Each of these metadata module descriptions was complete via the Pipeline module GUI and took about 30-45 minutes. As a demonstration, only the EMBOSS Matcher module is presented here.

▪ *Name*: EMBOSS_Matcher.pipe

▪ *URL*: http://www.loni.ucla.edu/twiki/bin/view/CCB/PipelineWorkflows_BioinfoEMBOSS

**Figure 6:**
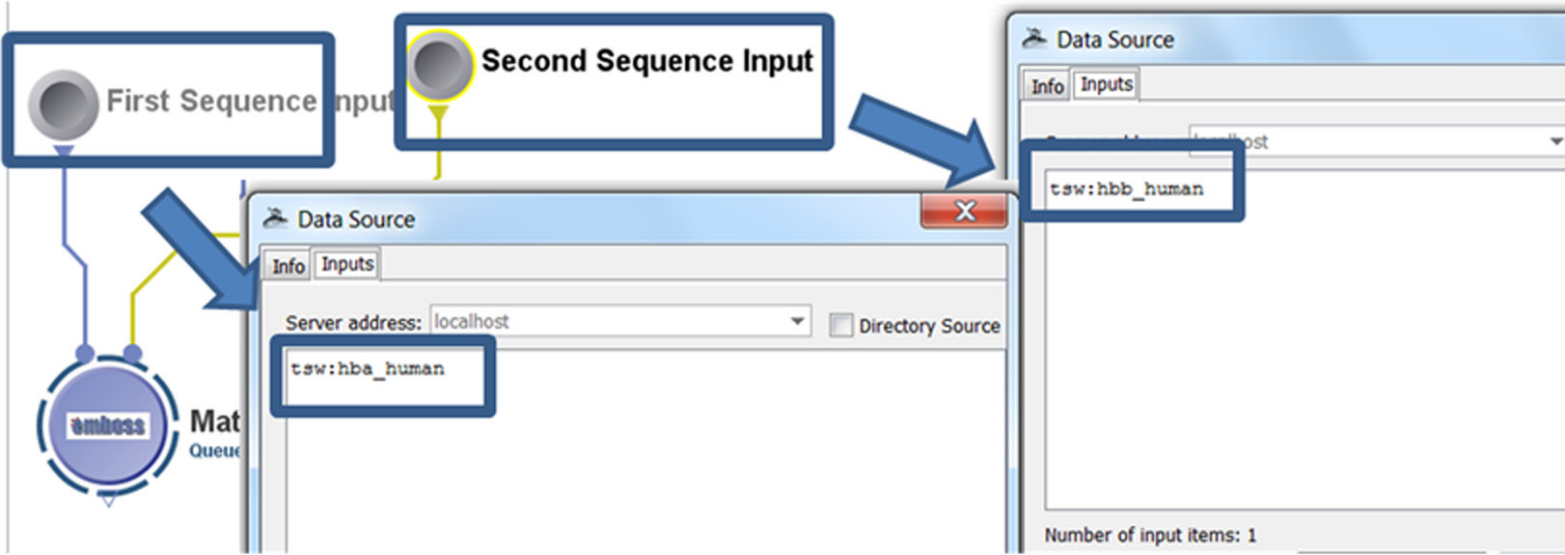
**A snapshot of the input parameters for the EMBOSS Matcher Pipeline workflow**.

**Figure 7:**
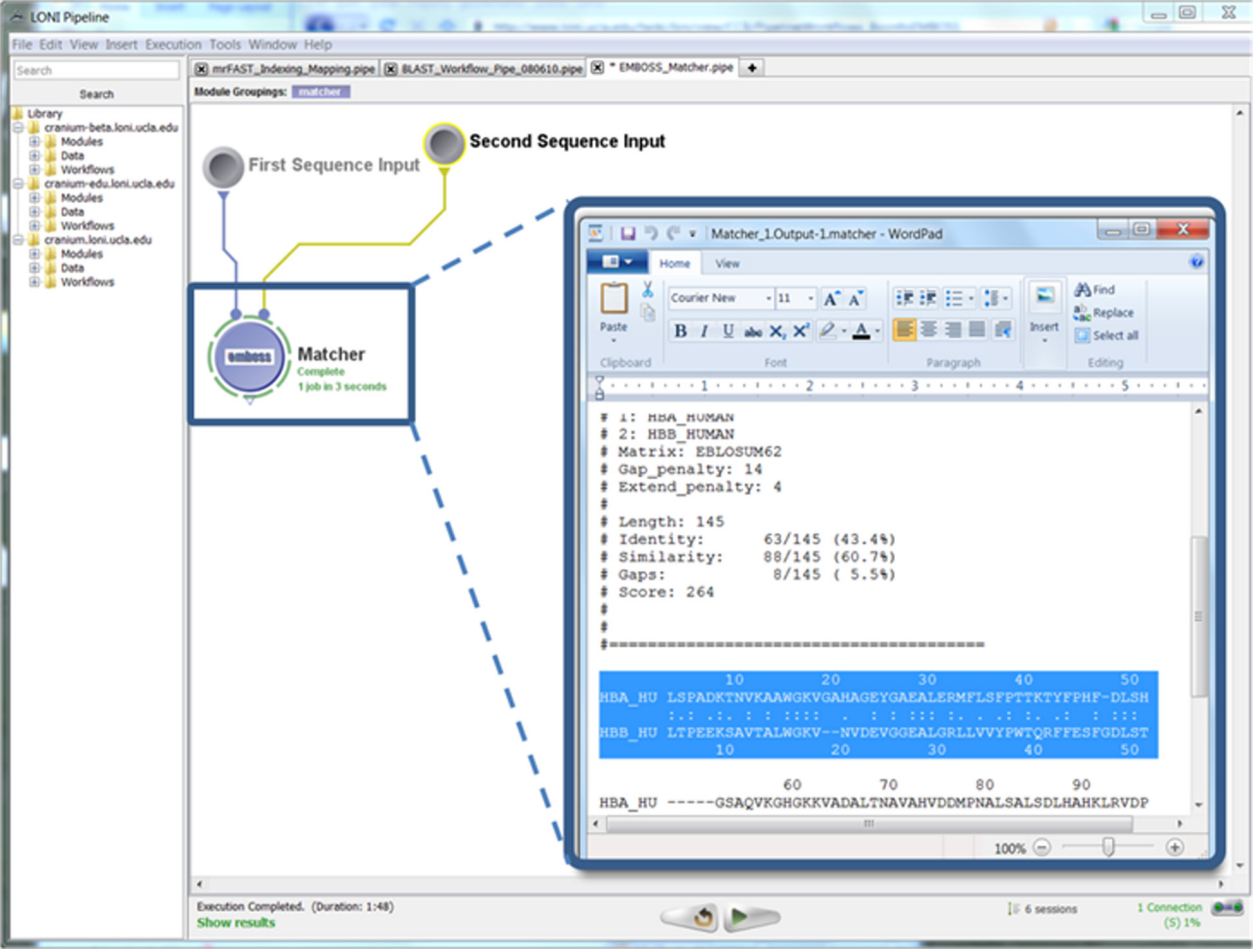
**A snapshot of the completed EMBOSS Matcher Pipeline workflow**. The Insert image shows the output result of the local sequence alignment of *hba_human *and *hbb_human*.

▪ *Screenshots*:

• Input: Figure 6 shows the input parameters (data-sources) for the corresponding Pipeline workflow.

• Pipeline Execution: Figure 7 demonstrates the completed execution of this EMBOSS module.

• Output: Table 4 contains the beginning of the output result from the EMBOSS Matcher alignment.

**Table 4:** A fragment of the text output of EMBOSS Matcher (see Figure 7)

########################################
# Program: matcher
# Rundate: Tue 2 Nov 2010 15:50:56
# Commandline: matcher
# -asequence tsw:hba_human
# -bsequence tsw:hbb_human
# -outfile/panfs/tmp/pipeline-edu/pipelnvr/2010November02_15h50m48s631ms/Matcher_1.Output-1.matcher
# Align_format: markx0
# Report_file:/panfs/tmp/pipeline-edu/pipelnvr/2010November02_15h50m48s631ms/Matcher_1.Output-1.matcher
#########################################

#=======================================
#
# Aligned_sequences: 2
# 1: HBA_HUMAN
# 2: HBB_HUMAN
# Matrix: EBLOSUM62
# Gap_penalty: 14
# Extend_penalty: 4

▪ *Approximate time to complete*: 2 minutes.

## III.3 mrFAST (micro-read Fast Alignment Search Tool)

◦ **URL**: http://mrFAST.sourceforge.net

◦ **Description**: mrFAST is designed to map short (micro) reads generated with the Illumina platform to reference genome assemblies in a fast and memory-efficient manner.

◦ **Installation**: The downloading, installation and configuration of the mrFAST suite on Linux OS kernel takes only 10 minutes following these instructions: http://mrfast.sourceforge.net/manual.html.

◦ **Pipeline Workflow**

▪ *XML Metadata description*: The example includes Pipeline module descriptions of the mrFAST Fasta-Indexing (indices can be generated in single or batch modes) and Fasta-Mapping (map single-end reads and paired-end reads to a reference genome) tools. Each of these metadata module descriptions was complete via the Pipeline module GUI and took about 10-15 minutes.

▪ *Name*: mrFAST_Indexing_Mapping.pipe

▪ *URL*: http://www.loni.ucla.edu/twiki/bin/view/CCB/PipelineWorkflows_BioinfoMRFAST

**Figure 8:**
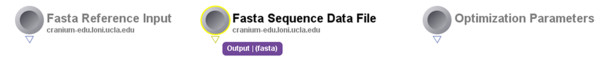
**A snapshot of the input parameters for the mrFAST Indexing Pipeline workflow**.

**Figure 9:**
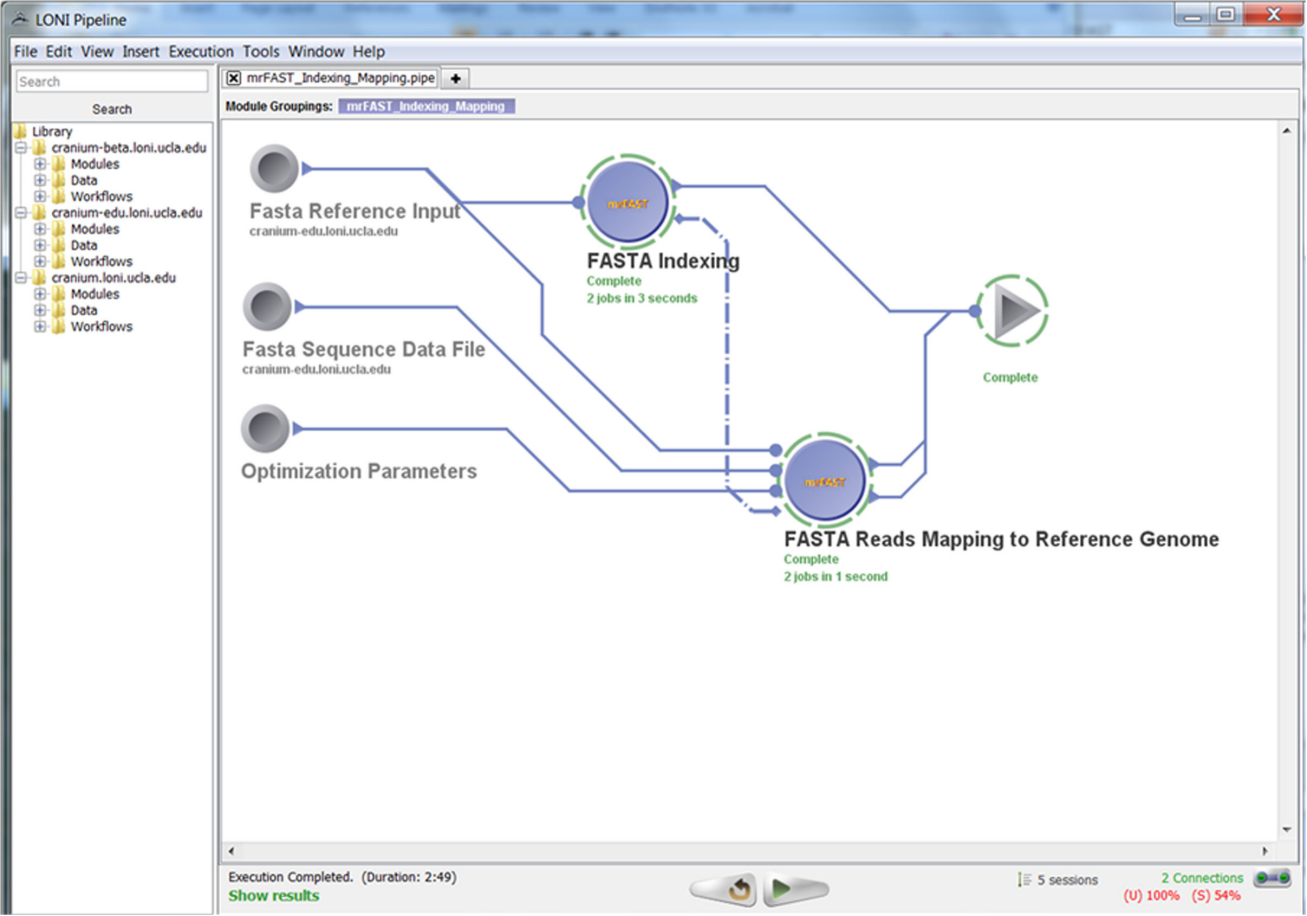
**A snapshot of the completed mrFAST Indexing Pipeline workflow**.

▪ *Screenshots*:

• Input: Figure 8 shows the input parameters (data-sources) for the corresponding Pipeline workflow.

• Pipeline Execution: Figure 9 demonstrates the completed execution of this mrFAST workflow.

• Output: Table 5 contains the beginning of the output result from the mrFAST workflow.

**Table 5:** A fragment of the text output of mrFAST Indexing workflow (see Figure 9)

**mrFAST unmapped output:**

>blue
CUGUCUGUCUUGAGACA

▪ *Approximate time to complete*: 2 minutes.

## III.4 GWASS

◦ **URL**: http://www.stats.ox.ac.uk/~marchini/software/gwas/gwas.html

◦ **Description**: The Genome-wide Association Study Software (GWASS) is a suite of tools facilitating the analysis of genome-wide association studies. These tools were used in the design and analysis of the 7 genome-wide association studies carried out by the Wellcome Trust Case-Control Consortium (WTCCC). One specific example of a GWASS tool is **IMPUTE **V.2 (IMPUTE2) which is used for genotype imputation and phasing https://mathgen.stats.ox.ac.uk/impute/impute_v2.html.

◦ **Installation**: The downloading, installation and configuration of the GWASS suite on Linux OS kernel takes about 15 minutes.

▪ *XML Metadata description*: Here we demonstrate the pipeline module description of Impute, which is a program for genotype imputation in genome-wide association studies and provides fine-mapping based on a dense set of marker data (such as HapMap).

▪ *Name*: GWASS_Impute.pipe

▪ *URL*: http://www.loni.ucla.edu/twiki/bin/view/CCB/PipelineWorkflows_BioinfGWASS

**Figure 10:**
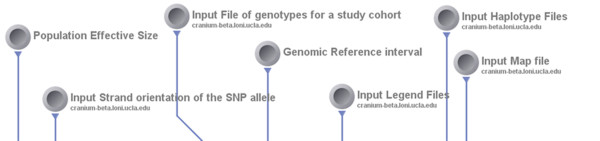
**A snapshot of the input parameters for the GWASS Impute Pipeline workflow**.

**Figure 11:**
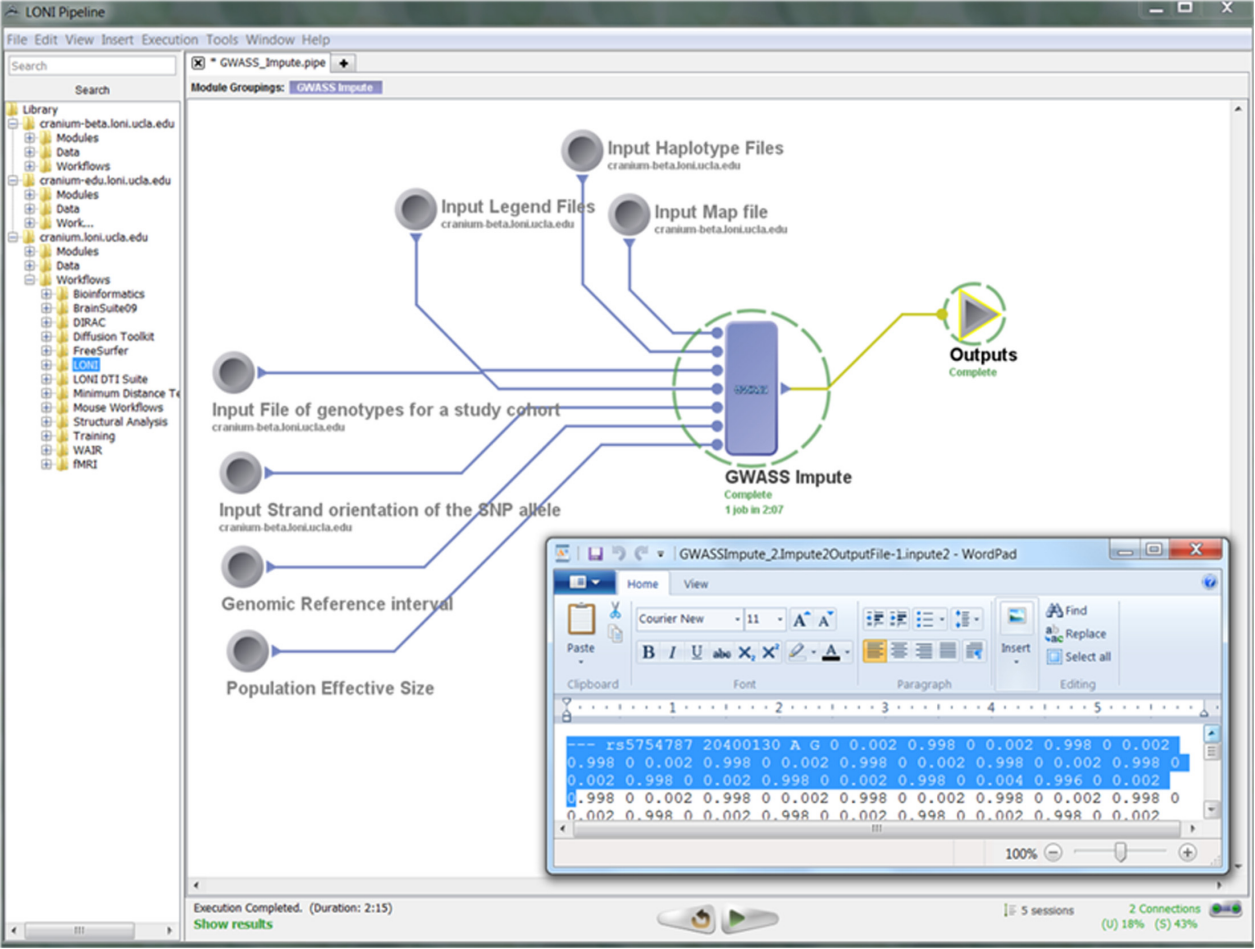
**A snapshot of the completed GWASS Impute Pipeline workflow**.

▪ *Screenshots*:

• Input: Figure 10 shows the input parameters (data-sources) for the corresponding Pipeline workflow.

• Pipeline Execution: Figure 11 demonstrates the completed execution of this GWASS Impute module.

• Output: Table 6 contains the beginning of the output result from the GWASS Impute module.

**Table 6:** A fragment of the text output of GWASS Impute workflow (see Figure 11)

---	rs5754787		20400130		A G	0	0.002	0.998	0	0.002	0.998	0	0.002	0.998	0
0	0.002	0.998	0	0.002	0.998	0	0.002	0.998	0	0.002	0.998	0	0.002	0.998	0
0	0.002	0.998	0	0.002	0.998	0	0.004	0.996	0	0.002	0.998	0	0.002	0.998	0
0	0.002	0.998	0	0.002	0.998	0	0.002	0.998	0	0.002	0.998	0	0.002	0.998	0
0	0.002	0.998	0	0.002	0.998	0	0.002	0.998	0	0.002	0.998	0	0.002	0.998	0
0	0.003	0.997	0	0.002	0.998	0	0.002	0.998	0	0.002	0.998	0	0.002	0.998	0
0	0.002	0.998	0	0.002	0.998	0	0.002	0.998	0	0.002	0.998	0	0.002	0.998	0
0	0.002	0.998	0	0.002	0.998	0	0.002	0.998	0	0.002	0.998	0	0.002	0.998	0
0	0.002	0.998	0	0.002	0.998	0	0.002	0.998	0	0.002	0.998	0	0.002	0.998	0
0	0.002	0.998	0	0.003	0.997	0	0.002	0.998	0	0.002	0.998	0	0.002	0.998	0
0	0.002	0.998	0	0.002	0.998	0	0.002	0.998	0	0.002	0.998	0	0.002	0.998	0
0	0.002	0.998	0	0.002	0.998	0	0.002	0.998	0	0.002	0.998	0	0.002	0.998	0
0	0.002	0.998	0	0.002	0.998	0	0.002	0.998	0	0.003	0.997	0	0.002	0.998	0
0	0.002	0.998	0	0.002	0.998	0	0.002	0.998	0	0.002	0.998	0	0.002	0.998	0
0	0.002	0.998	0	0.002	0.998	0	0.002	0.998	0	0.002	0.998	0	0.002	0.998	0
0	0.002	0.998	0	0.002	0.998	0	0.002	0.998	0	0.002	0.998	0	0.002	0.998	0
0	0.002	0.998	0	0.002	0.998	0	0.002	0.998	0	0.002	0.998	0			
...															

▪ *Approximate time to complete*: 2-3 minutes.

## III.5 Interoperabilities between independently developed informatics resources

**Figure 12:**
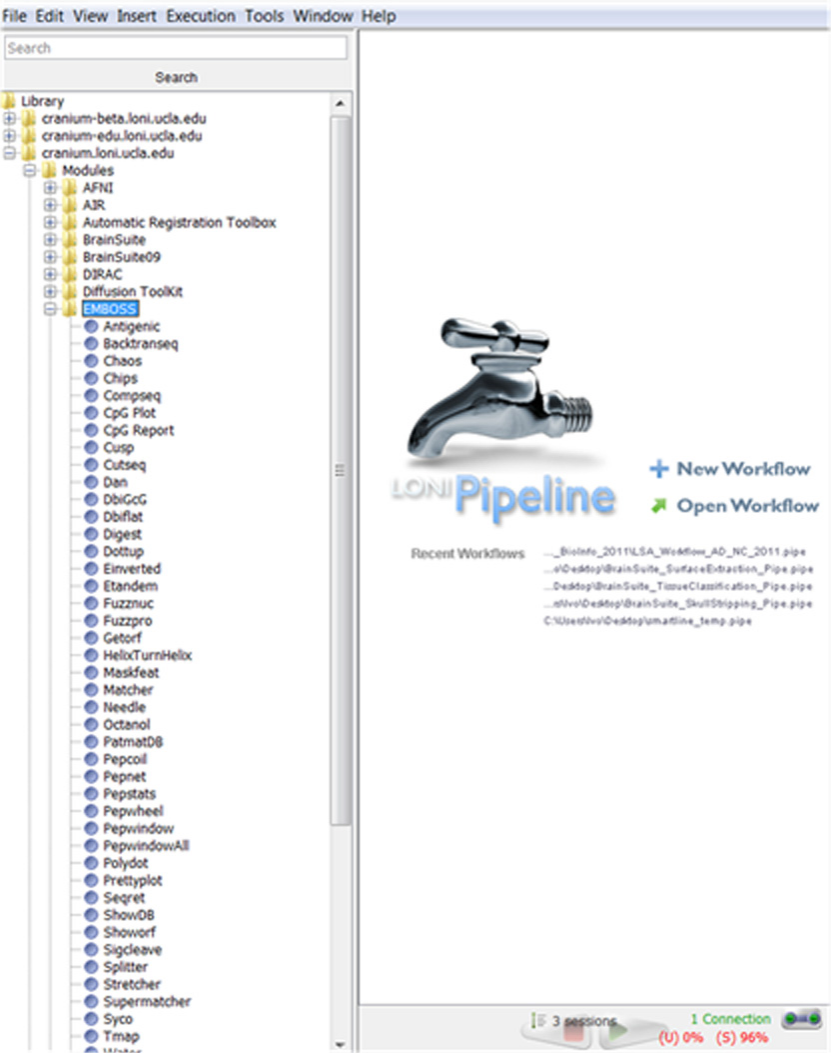
**Pipeline Server Library**.

There are 100's of examples of heterogeneous Pipeline graphical workflows that illustrate the interoperability between imaging tools independently developed for different purposes by different investigators at remote institutions. These can be found under the Workflows section of the Pipeline library, Figure 12, as well as on the web at: http://www.loni.ucla.edu/twiki/bin/view/LONI/Pipeline_GenomicsInformatics.

Similarly, bioinformatics and genomics investigators can construct novel computational sequence analysis protocols using the available suites of informatics resources (data, tools and services). Below is one example of integrating the informatics tools described above.

### III.5.1 Indexing and Mapping

In this example, we illustrate how to integrate mrFAST and EMBOSS Water informatics tools. The left branch of the Pipeline workflow uses EMBOSS to align 2 sequences (**tsw:hba_human **&**tsw:hbb_human**) using Water. The right branch of the workflow does Fast Alignment using 2 independent datasets (EMBOSS Water-computed: **Water_1.Output-1.fasta **& mrFAST:**cofold-blue.fasta**) and 2 separate mrFAST references (**query.fasta **&**dna.fasta**). The interesting demonstration here is that the EMBOSS Water output (of the **tsw:hba_human **&**tsw:hbb_human **registration) is later directly used as *derived data sequence *which is re-aligned to the 2 mrFAST reference sequences.

◦ Pipeline Workflow

▪ *Design*: This tool-interoperability example illustrates feeding the output of the EMBOSS Water module (fasta file) as in input in mrFAST Indexing module and subsequent mapping using mrFAST.

▪ *URL*: http://www.loni.ucla.edu/twiki/bin/view/CCB/PipelineWorkflows_BioinfoMRFAST

▪ *Name*: BioInfo_IntegratedWorkflow_EMBOSS_Water_mrFAST.pipe

**Figure 13:**
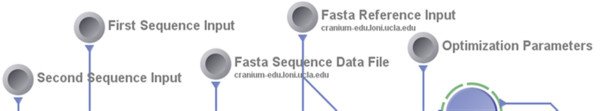
**A snapshot of the input parameters for this heterogeneous Pipeline workflow**: EMBOSS: tsw:hba_human, tsw:hbb_human mrFAST: cofold-blue.fasta, query.fasta, dna.fasta.

**Figure 14:**
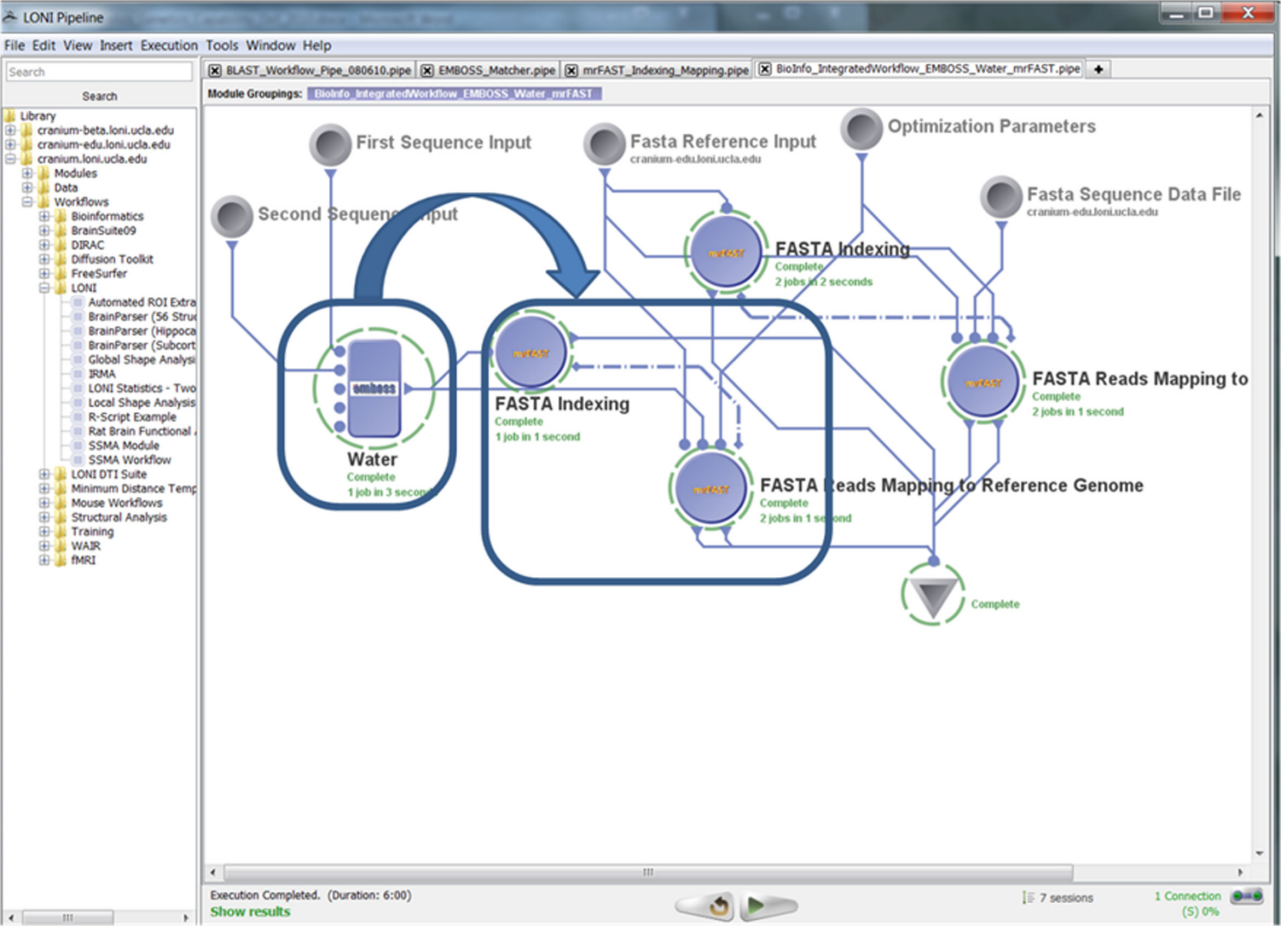
**A snapshot of the completed heterogeneous (EMBOSS/mrFAST) Pipeline workflow**.

▪ *Screenshots*:

• Inputs: Figure 13 shows the input parameters (data-sources) for the corresponding Pipeline workflow.

• Pipeline Execution: Figure 14 demonstrates the completed execution of this EMBOSS/mrFAST workflow.

▪ Output: Table 7 contains the beginning of the output result from this heterogeneous workflow.

**Table 7:** A fragment of the text output of this heterogeneous pipeline workflow (see Figure 14)

**FASTAReadsMappingtoReferenceGenome_2.OutputUnmapped-1.map**
>HBA_HUMAN P69905 Hemoglobin subunit alpha (Hemoglobin alpha chain) (Alpha-globin)
LSPADKTNVKAAWGKVGAHAGEYGAEALERMFLSFPTTKTYFPHF-DLS-----HGSAQV
>GHGKKVADALTNAVAHVDDMPNALSALSDLHAHKLRVDPVNFKLLSHCLLVTLAAHLPA
EFTPAVHASLDKFLASVSTVLTSKY
>HBB_HUMAN P68871 Hemoglobin subunit beta (Hemoglobin beta chain) (Beta-globin) (LVV-hemorphin-7)
LTPEEKSAVTALWGKV--NVDEVGGEALGRLLVVYPWTQRFFESFGDLSTPDAVMGNPKV
>AHGKKVLGAFSDGLAHLDNLKGTFATLSELHCDKLHVDPENFRLLGNVLVCVLAHHFGK
EFTPPVQAAYQKVVAGVANALAHKY

▪ *Approximate time to complete*: 6 minutes.

### III.5.2 Alignment and Assembly

Another interesting example illustrating the power of tool interoperability using the Pipeline environment involved the Alignment and Assembly computational genomics protocol we presented in the **Implementation **section. In this example, we demonstrate the interoperability between MAQ, SAMtools, and Bowtie tools.

◦ Pipeline Workflow

▪ *Design*: This genomics pipeline workflow begins with an optional preprocessing step extracting a sub-sequence of the genomic sequence (for both the forward and reverse sequences). Then, the Illumina sequencing data (sequence.txt) are converted first to fastq and later (together with the reference genome fasta file) to binary fastq file (bfq) format. The data is then aligned to a reference genome, the MAP file is converted to BAM file and the reference genome is indexed. The alignment MAP file is converted to SAMtools (SAM) format first and then to a binary BAM file. Next, duplicated reads are removed, the BAM file is sorted, MD tagged and indexed. In addition the MAQ-based alignment, Bowtie was used (in parallel) to align the data to the reference sequence. Such alternative paths in the data processing protocol are easily constructed and modified in the Pipeline environment.

▪ *URL*: http://www.loni.ucla.edu/twiki/bin/view/CCB/PipelineWorkflows_BioinfoMAQ

▪ *Name*: MAQ_SAMtools_Bowtie_Integrated_Cranium.pipe

**Figure 15:**
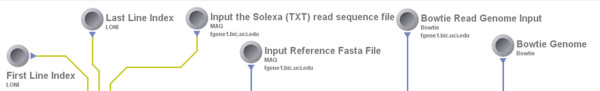
**A snapshot of the input parameters for this heterogeneous Pipeline workflow**.

**Figure 16:**
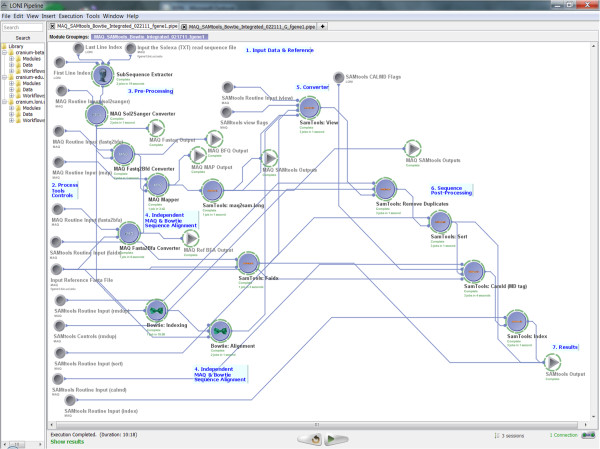
**A snapshot of the completed heterogeneous Pipeline workflow**. The image shows the expanded (raw, unfolded) version of the protocol which is analogous to the folded version of the same pipeline workflow illustrated on Figure 3. The folded version only demonstrates the major steps in the protocol and abstracts away some of the technical details, however, both versions of this protocol perform identical analyses.

▪ *Screenshots*:

• Inputs: Figure 15 shows the input parameters (data-sources) for the corresponding Pipeline workflow.

• Pipeline Execution: Figure 16 demonstrates the completed execution of this heterogeneous pipeline workflow.

▪ Output: Table 8 contains the beginning of the output result from this heterogeneous workflow.

**Table 8:** A fragment of the text output of this heterogeneous pipeline workflow (see Figure 16)

**SamToolsCamldMDtag_1.OutputNo-DuplicatesBAMfile-1.bam.bai (Binary file)**			
**SamToolsCamldMDtag_1.OutputNo-DuplicatesBAMfile-1.bam (Binary file)**			
...			
**BowtieEcoli_1.OutputMAPfile-1.map (ASCII file)**			
r0	- gi|110640213|ref|NC_008253.1|	3658049	
	ATGCTGGAATGGCGATAGTTGGGTGGGTATCGTTC		
	45567778999:9;;<===>?@@@@AAAABCCCDE	0	32:T>G,34:G > A
r1	- gi|110640213|ref|NC_008253.1|	1902085	
	CGGATGATTTTTATCCCATGAGACATCCAGTTCGG		
	45567778999:9;;<===>?@@@@AAAABCCCDE	0	
r2	- gi|110640213|ref|NC_008253.1|	3989609	
	CATAAAGCAACAGTGTTATACTATAACAATTTTGA		
	45567778999:9;;<===>?@@@@AAAABCCCDE	0	
r5	+ gi|110640213|ref|NC_008253.1|	4249841	
	CAGCATAAGTGGATATTCAAAGTTTTGCTGTTTTA		
	EDCCCBAAAA@@@@?>===<;;9:99987776554	0	
...			

▪ *Approximate time to complete*: 4-5 minutes.

## IV. Conclusions

This paper reports on the applications of the Pipeline environment [15,34] to address two types of computational genomics and bioinformatics challenges - graphical management of diverse suites of tools, and the interoperability of heterogeneous software. Specifically, this manuscript presents the concrete details of deploying general informatics suites and individual software tools to new hardware infrastructures, the design, validation and execution of new visual analysis protocols via the Pipeline graphical interface, and integration of diverse informatics tools via the Pipeline XML syntax. We demonstrate each of these three protocols using several established informatics packages - miBLAST (a tool for efficient Basic Local Alignment Search Tool of a batch of nucleotide sequence queries), EMBOSS (European Molecular Biology Open Software Suite), mrFAST (micro-read Fast Alignment Search Tool), GWASS (Genome-wide Association Study Software), MAQ (Mapping and Assembly with Qualities), SAMtools (Sequence Alignment and Mapping Tools), and Bowtie. These examples demonstrate informatics and genomics applications using the Pipeline graphical workflow environment. The Pipeline is a platform-independent middleware infrastructure enabling the description of diverse informatics resources with well-defined invocation protocol - syntax for dynamic specification of the inputs, outputs and run-time control parameters. These resource XML descriptions may be provided using the Pipeline graphical user interface or using machine code according to the Pipeline XSD schema.

The Pipeline environment provides a powerful and flexible infrastructure for efficient biomedical computing and distributed informatics research. The interactive Pipeline resource management enables the utilization and interoperability of diverse types of informatics resources. The Pipeline client-server model provides computational power to a broad spectrum of informatics investigators - experienced developers and novice users, the computational-resources haves and have-nots, as well as between basic and translational scientists. The open development, validation and dissemination of computational networks (pipeline workflows) facilitates the sharing of knowledge, tools, protocols and best practices, and enables the unbiased validation and replication of scientific findings by the entire community.

For specific research domains, applications and needs, there are pros and cons for using the Pipeline environment or any of the alternative tools and infrastructures for visual informatics and computational genomics. Examples of powerful alternatives include Taverna [35], Kepler [14], Triana [36], Galaxy [37], AVS [38], VisTrails [39], Bioclipse [40], KNIME [41], and others. The main advantages of the Pipeline environment are the distributed client-server architecture with diverse arrays of grid plug-ins, the lightweight data, tools and services utilization and the dynamic workflow design, validation, execution, monitoring and dissemination of complete end-to-end computaitonal solutions.

## V. Availability and requirements

• **Project name**: Pipeline Environment

• Project home pages:

◦ LONI: http://pipeline.loni.ucla.edu

◦ NITRC: http://www.nitrc.org/projects/pipeline

◦ BIRN: http://www.birncommunity.org/tools-catalog/loni-pipeline

◦ Bioinformatics.org: http://www.bioinformatics.org/pipeline

◦ Try the LONI Pipeline Informatics and Genomics Workflows online without any software installation using anonymous guest account: http://pipeline.loni.ucla.edu/PWS

• **Operating system(s)**: Pipeline **clients **and **servers **are platform-independent, while some features (e.g. privilege escalation, failover) require the server run on Linux/UNIX OS. The Distributed Pipeline Server (DPS) graphical user interface, which installs the Pipeline server, Grid Engine, and computational imaging and informatics software tools, require standard Linux OS kernels. The Pipeline Web Start (PWS) allows users to start the Pipeline application directly from the web browser and run it locally without any installation. It has all the features and functionality of the downloadable stand-alone Pipeline application and allows anonymous guest access or user-authentication to connect to remote Pipeline servers.

• **Programming language**: Pure Java.

• **Other requirements:**

◦ Requirements Summary: The Pipeline client and server can run on any system that is supported by Java Runtime Environment (JRE) 1.5 or higher. Windows Pipeline servers will not be able to use privilege escalation. Three-tier Failover feature is only supported by Unix/Linux systems. All other features are available for all platforms. Most Distributed Pipeline Servers require 300-1,000MB memory, which may depend on the load and garbage collection preferences.

◦ For distributed multicore deployment, the Distributed Pipeline Server (DPS) requires a Grid manager (e.g., Distributed Resource Management Application API, DRMAA), which is provided with the DPS distribution. The Pipeline server will still work on a platform without a Grid manager, however, jobs may not be processed in parallel and performance on multicore machines may be suboptimal.

◦ Complete requirements:

▪ Client: http://pipeline.loni.ucla.edu/support/user-guide/installation/

▪ Server: http://pipeline.loni.ucla.edu/support/server-guide/installation/

▪ DPS: http://pipeline.loni.ucla.edu/DPS

▪ PWS: http://pipeline.loni.ucla.edu/PWS

• **License**: Apache-derived software license http://www.loni.ucla.edu/Policies/LONI_SoftwareAgreement.shtml.

• **Caution**: There are some potential limitations of the Pipeline environment and its current collection of data, tools services and computational library (module XML meta-data descriptions):

◦ Each new informatics tool which needs to be accessible as a processing module within the Pipeline environment needs to be **described manually by an expert using the Pipeline GUI or automatically using a properly configured XML exporter **(e.g., http://www.loni.ucla.edu/twiki/bin/view/MAST/TranslateTo). Then the Pipeline XML module description can be shared with other users.

◦ To run available Pipeline workflows (*.pipe workflow files) on **remote Pipeline-servers**, users need to have accounts on the remote Pipeline servers. In addition, 2 types of updates may be necessary in the PIPE files - the server-name references of data sources (inputs), data sinks (results), and executables, as well as the path references to the data sources, sinks and executables. The server-name can be easily updated using server changer tool in Pipeline (Tools menu → Server Changer). User has to edit path references on some or all of the data sources, sinks and executables for their server. No workflow modifications are necessary for executing these pipeline workflows on the LONI Pipeline Cranium server; however this requires a LONI Pipeline user-account http://www.loni.ucla.edu/Collaboration/Pipeline/Pipeline_Application.jsp. A proper administrator configuration of the Distributed Pipeline Server (DPS, http://pipeline.loni.ucla.edu/DPS) will resolve the need for such revisions by the user.

◦ Some computational tools **may require wrapper scripts **that call the raw executable binaries. These scripts (not the raw binaries) are then invoked via the Pipeline environment. Example situations include tools that have implicit outputs, or if the tools routinely return non-trivial exit codes, distinct from zero. Such problems may cause the Pipeline environment to halt execution of subsequent modules, because of a broken module-to-module communication protocol.

◦ Smartlines, which **auto-convert between different informatics data formats**, need to be extended to handle informatics and genomics data (currently, smartlines handle mostly image file format conversions).

◦ **Access to external informatics databases **may need to be customized - e.g., PDB http://www.rcsb.org, SCOP http://scop.mrc-lmb.cam.ac.uk/scop, GenBank http://www.ncbi.nlm.nih.gov/genbank, etc.

◦ Native vs. Virtual Pipeline server: The fully **distributed pipeline server **(**DPS**) architecture (which allows anyone to locally download, configure and deploy the complete Pipeline server) provides (natively) both the Pipeline middleware as well as installers for all computational tools available on the LONI Cranium Pipeline Grid Server http://pipeline.loni.ucla.edu/support/user-guide/interface-overview/. The virtual Pipeline server and pipeline clients also provide the complete Pipeline environment for a virtual VMware invocation http://pipeline.loni.ucla.edu/PNVE.

• **Any restrictions to use by non-academics**: Free for non-commercial research purposes.

## Competing interests

The authors declare that they have no competing interests.

## Authors' contributions

All authors have read and approved the final manuscript. All authors made substantive intellectual contributions to this research and development effort and the drafting of the manuscript. Specifically, the authors contributed as follows: IDD article conception, study design, workflow implementation and applications, data processing, drafting and revising the manuscript; FT study design, workflow implementation, data processing, drafting the manuscript; FM study design, workflow implementation, drafting the manuscript; PP pipeline software implementation, workflow design and drafting the manuscript; ZL pipeline software implementation, workflow design and drafting the manuscript; AZ pipeline software implementation, study design, workflow development and drafting the manuscript; PE pipeline software engineering, workflow design and drafting the manuscript; JP pipeline server Grid implementation and management, workflow development and drafting the manuscript; AG study design, workflow implementation and drafting the manuscript, JAK and APC study design protocol, data processing, workflow development and drafting the manuscript; JDVH study design protocol and drafting the manuscript; JA workflow applications and drafting the manuscript; CK study design, workflow applications and drafting the manuscript; AWT study design, engineering of the pipeline environment, and drafting the manuscript.
